# Editorial: Management of peritoneal surface malignancies. (cytoreductive surgery, HIPEC, PIPAC, and beyond)

**DOI:** 10.3389/fonc.2024.1340737

**Published:** 2024-03-12

**Authors:** Amine Souadka, Naoual Bakrin

**Affiliations:** ^1^ Surgical Oncology Department, National Institute of Oncology, University Mohammed V in Rabat, Rabat, Morocco; ^2^ Centre d’Investigation Clinique, Centre Hospitalier Universitaire Ibn Sina, Rabat, Morocco; ^3^ Equipe de Recherche en Oncologie Transationelle (EROT), University Mohammed V in Rabat, Rabat, Morocco; ^4^ Department of Surgical Oncology, Hospices Civils de Lyon, Centre Hospitalier Lyon-Sud, Lyon, France; ^5^ Centre pour l’Innovation en Cancérologie de Lyon (CICLY), Claude Bernard University Lyon 1, Lyon, France

**Keywords:** peritoneal surface malignancies, HIPEC (heated intraperitoneal chemotherapy), cytoreductive surgery (CRS), PIPAC, molecular status

Managing patients afflicted with peritoneal metastases stands as a formidable challenge within the realm of oncology. These individuals grapple with significant tumor growth that infiltrates multiple abdominal organs, leading to a spectrum of symptoms ranging from mild discomfort and early satiety to more severe complications such as ascites, bowel obstruction, and a drastic decline in their overall quality of life.

In recent years, strides in innovative systemic therapies, surgical techniques, and patient selection criteria have markedly improved outcomes for those facing this dire condition. (Mangieri and Levine) The focal point of this Research Topic lies in a comprehensive exploration of peritoneal metastasis treatment, spanning from the broader context of public health, (Aquina et al.) to the intricate molecular levels of intervention. (Breusa et al.).

Cytoreductive Surgery (CRS) and Hyperthermic Intraperitoneal Chemotherapy (HIPEC) have transitioned from being niche procedures offered by a select group of dedicated surgeons and institutions to becoming globally accessible techniques. The trend towards standardization, exemplified by collaborative efforts such as the study by Bhatt et al., (1) has ushered in a new era in the treatment landscape. Over the past two decades, the number of CRS/HIPEC-performing surgeons has significantly increased worldwide. However, disparities in care provision persist, as evidenced by Aquina et al‘ s investigation into patient access in the United States and Tan et al’s study on the incidence and outcomes of delayed treatment of peritoneal metastasis in Singapore.

While the effectiveness of systemic therapies has been substantiated through rigorous phase III randomized trials, evidence supporting CRS/HIPEC primarily stems from extensive retrospective studies and consensus among expert groups. Notably, CRS/HIPEC has demonstrated remarkable efficacy in managing rare tumor types like appendix and mesothelioma. Ovarian cancer occupies a pivotal position in this narrative, emerging as a quintessential example of peritoneal surface malignancy. (2) Despite initial resistance from influential figures, it has gained widespread acceptance and found its place in prominent guidelines such as NCCN, ESGO, and the French Guidelines for irresectable disease (3). Recent international collaborative endeavors, such as the Consolidation HIPEC study (CHIPOR), have underscored a substantial survival advantage, particularly in patients with previously platinum-exposed disease. (4) The molecular rationale underpinning this benefit lies in hyperthermia’s role, impairing homologous recombination and DNA replication—a topic thoroughly explored by Breusa et al‘s review on the molecular rationality of locoregional approaches in ovarian cancer. (5). Additionally, the critical question of secondary cytoreductive surgery for recurrent ovarian cancer, as raised by de Bree et al adds depth to our understanding of this evolving field.

Encouragingly, recent surgical outcome data affirm the safety of CRS/HIPEC, aligning it with routinely performed complex oncologic surgeries. This approach has been formally integrated into surgical oncology fellowship training programs, reflecting its growing significance. One notable area of focus revolves around early detection of postoperative complications and the subsequent development of specialized center procedures to rescue patients—a testament to the evolving expertise in this domain.

In navigating this multifaceted terrain of peritoneal metastases, collaborative efforts, innovative techniques, and a relentless commitment to research underscore our progress. As we delve deeper into understanding the molecular intricacies and refine our surgical approaches, the collective aim remains steadfast: to offer not just treatment, but genuine hope and improved quality of life to those bravely battling this complex condition.

Amidst the revolutionary landscape of peritoneal surface malignancy management, a groundbreaking technique has emerged, promising a paradigm shift in the way we approach treatment: Pressurized Intraperitoneal Aerosol Chemotherapy (PIPAC). While still in its developmental phase, PIPAC represents a beacon of hope, offering a highly targeted and minimally invasive alternative for patients battling peritoneal surface metastases (PSM). The ongoing trials and research endeavors surrounding PIPAC signify a promising future, where patients might experience treatments characterized by fewer side effects and quicker recovery times.

A recent retrospective cohort study, (Kefleyesus et al.) conducted across 18 international centers, delved into the realm of PIPAC as a treatment modality for peritoneal surface metastases originating from recurrent or progressive ovarian cancer (OC).

Remarkably, the study demonstrated low morbidity and mortality rates associated with PIPAC, affirming its safety as a palliative treatment option. The promising outcomes observed after three PIPAC cycles not only validated its efficacy but also hinted at the possibility of refining treatment strategies to optimize patient outcomes further.

Looking ahead, collaborative efforts, rigorous research, and a commitment to refining our understanding of molecular intricacies are paramount. By delving deeper into the molecular underpinnings and continuously refining our surgical techniques, we are not merely offering treatment but also genuine hope and an improved quality of life to those bravely facing peritoneal surface metastases. The ongoing trials and research endeavors surrounding PIPAC, along with the evolution of established treatments like CRS/HIPEC, underscore our collective dedication to advancing the field. As we navigate this multifaceted terrain, our goal remains steadfast: to enhance not just survival rates but the overall well-being and resilience of patients battling this complex condition.

As we chart the course of progress in the management of peritoneal metastases, numerous unresolved questions emerge, casting a spotlight on the intricacies and challenges inherent in this field. First and foremost, patient selection remains a critical yet ambiguous area, where defining the ideal candidate for advanced interventions like CRS, HIPEC, and PIPAC continues to elude consensus. Additionally, the nuances of complete cytoreductive surgery (CRS) and the optimal parameters for Hyperthermic Intraperitoneal Chemotherapy (HIPEC) present a complex puzzle, one that intertwines surgical precision with therapeutic efficacy. The evolving landscape of Pressurized Intraperitoneal Aerosol Chemotherapy (PIPAC) adds another layer to this multifaceted scenario, promising a less invasive yet equally potent approach but leaving us with questions about its long-term outcomes and optimal application. Furthermore, as we delve into the realm of drug regimens, immunotherapy, and molecular profiling, we confront a myriad of uncertainties about the best treatment combinations, patient-specific therapies, and the molecular underpinnings that could guide our clinical decisions (**Figure 1**).

**Figure 1:**
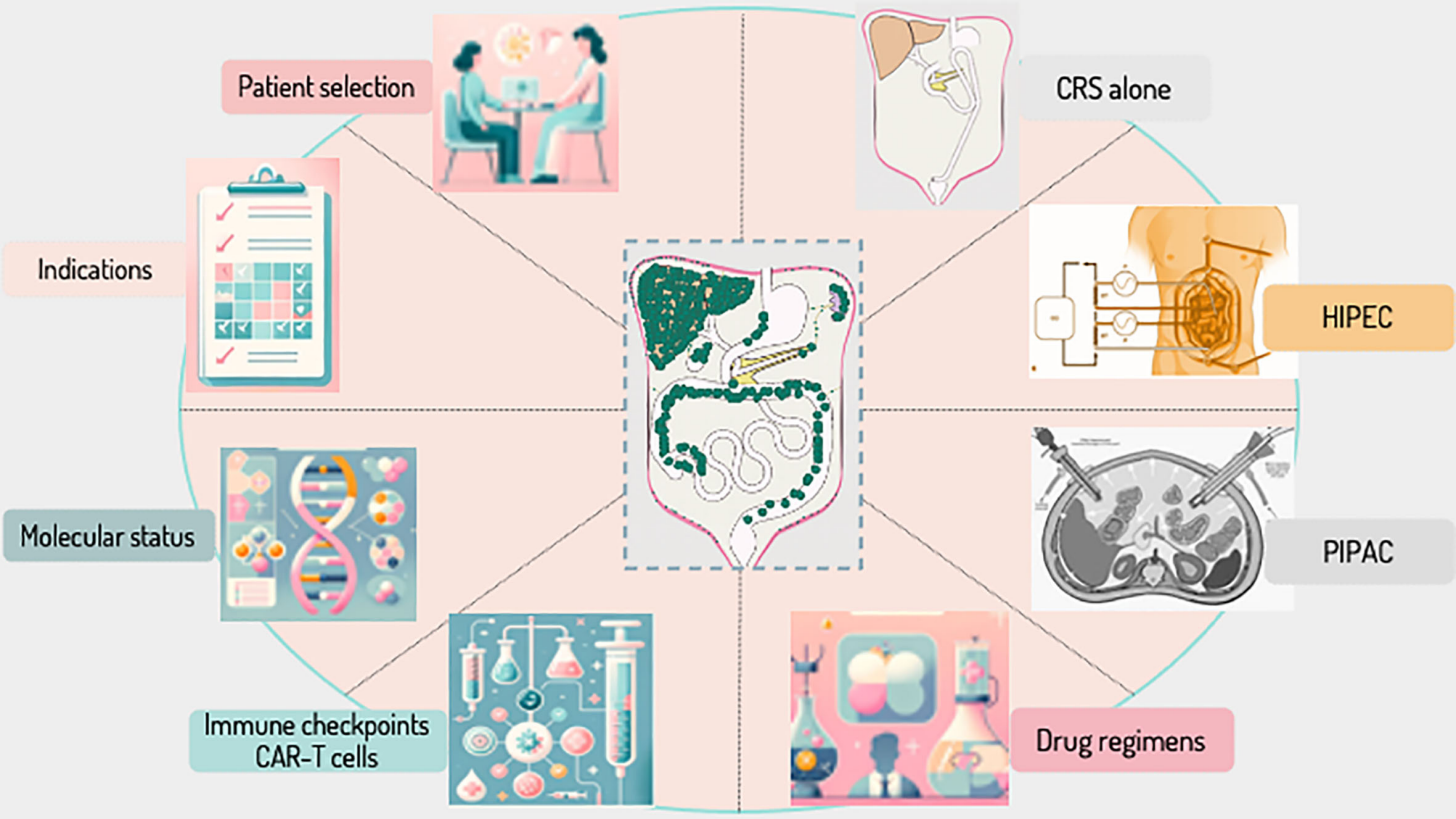
Navigating the Uncharted Waters of Peritoneal Surface Malignancy Management – Key Unresolved Questions. This figure delineates the principal uncertainties and ongoing debates in the treatment of peritoneal surface malignancies. It encompasses a spectrum of critical aspects, from patient selection to the intricacies of surgical and chemotherapeutic interventions. Each segment reflects a pivotal area of inquiry: **Patient Selection**: The enigma of creating robust, universally applicable criteria for patient eligibility for advanced treatments. **Complete Cytoreductive Surgery**: The challenge in defining and achieving complete cytoreduction, and the quest for predictive accuracy in preoperative evaluations. **HIPEC**: The pursuit of standardizing Hyperthermic Intraperitoneal Chemotherapy parameters to balance efficacy and safety. **PIPAC**: The exploration of long-term efficacy and safety profiles for Pressurized Intraperitoneal Aerosol Chemotherapy. **Drug Regimen**: The conundrum of optimizing chemotherapeutic agents and regimens tailored to individual patient and tumor profiles. **CAR T-Cells and Checkpoint Inhibitors**: The exploration into identifying predictive markers for responsiveness to CAR T cells and checkpoint inhibitors in the treatment of peritoneal surface malignancies. **Molecular Status**: The challenge of integrating molecular diagnostics into personalized treatment strategies. **Indication of Each Procedure**: The ongoing debate over the specific indications and comparative effectiveness of various treatment modalities.

In conclusion, as we stand at the intersection of innovative therapies and evolving surgical approaches, the future of peritoneal surface malignancy management holds the promise of personalized, precise, and compassionate care. Our journey is far from over; instead, it is a continuum of discovery and dedication, fueled by the shared vision of a future where peritoneal metastases are not just treatable but conquerable, and where every patient receives the best care possible, regardless of their geographical location or socioeconomic status.

## Author contributions

AS: Writing – original draft, Writing – review & editing. NB: Writing – original draft, Writing – review & editing.
